# Social Context Modulates Facial Imitation of Children’s Emotional Expressions

**DOI:** 10.1371/journal.pone.0167991

**Published:** 2016-12-08

**Authors:** Peter A. Bos, Nadine Jap-Tjong, Hannah Spencer, Dennis Hofman

**Affiliations:** Department of Experimental Psychology, Utrecht University, Utrecht, The Netherlands; Universita degli Studi di Pisa, ITALY

## Abstract

Children use emotional facial expressions of others for guiding their behavior, a process which is important to a child’s social-emotional development. Earlier studies on facial interaction demonstrate that imitation of emotional expressions of others is automatic, yet can be dynamically modulated depending on contextual information. Considering the value of emotional expressions for children especially, we tested whether and to what extent information about children’s temperament and domestic situation alters mimicry of their emotional expressions. Results show that angry expressions of children displaying negative behavior resulted in stronger imitation, which may serve as a corrective signal. Sad facial expressions resulted in stronger imitation towards those behaving positively but only when exposed to a difficult domestic situation, indicating increased empathy towards these children. These findings shed new light on the dynamic implicit communicative processes that shape interaction with children of different social-emotional backgrounds.

## Introduction

Emotional facial expressions serve a critical communicative function in human social interaction [1]. In the evolutionary development of modern humans, facial expressions in combination with gestural communication most certainly predate language as the key medium to transmit information and facilitate mutual understanding in dyadic interaction [2]. A key communicative function of emotional facial expressions is the signaling of either corrective of affiliative normative values. Especially during ontogenetic development, facial expressions towards children are an effective means to guide their behavior [3]. Infants are extremely sensitive to the emotional expressions of their caregivers, as indicated by altered neural responses to adult emotional facial expressions and prosody in 7-month-old infants [4]. Furthermore, a classical study showed that one-year-old children either crossed or did not cross a visual cliff depending on the facial expression of their mothers [5]. Whereas joyful or interested facial expressions motivated children to cross a visual cliff, a mother’s facial expression of fear or anger refrained the children from crossing [5]. This process, whereby children use emotional expressions of others to guide and regulate their behavior is known as social referencing [6, 7], and it is thought to contribute to children’s socialization when growing up [8]. For example, emotional responses of caregivers to children’s emotions, and the caregivers own level of emotional expression affect a child’s development of emotion regulation and social competence [8]. This illustrates the importance of reciprocal facial communication between child and caregiver throughout development [7].

Research investigating interpersonal interaction of emotional facial expressions, mostly using electromyography (EMG), has demonstrated such communication to be rapid, automatic, and largely unconscious [9, 10]. Overall, observing emotional facial expressions in others elicits motor activation involved in the production of the expression observed [10]. This automatic imitation of facial expressions is termed ‘facial mimicry’ and has also been observed in primate species such as orangutans, macaques, and geladas [11–13]. In geladas, facial mimicry is predicative of the duration of social play behavior [14], which is in line with evolutionary models that stress the importance of perception-action coupling in underlying social synchrony and affiliation [15]. In humans, facial mimicry is thought to aid empathic processes and emotion recognition [16, 17]. While mimicry of happy faces can serve as a signal of social affiliation, increased frowning can result from different underlying motives [10]. Frowning, characterized by activation of the corrugator supercilii muscle above the eye, is a critical component of the basic facial expressions of anger and sadness [18] and has, for example, been related to a negative mood, negative attitudes towards a presented stimulus, concentration [17], as well as empathic concern [19]. As such, mimicry of frowning in response to either angry of sad facial expression most likely reflects different motivations, depending on the context. Frowning in response to sadness in others has been related to sympathy and empathic behavior towards others [19]. A series of studies in adults and children shows increased frowning especially towards sadness inducing stimuli, which is predictive of subsequent prosocial behavior [19, 20]. Also, compared to controls, adolescents with disruptive behavioral disorder that score high on callous-unemotional traits, give lower subjective ratings of empathy toward sadness inducing stimuli. An effect that is accompanied by reduced activation of the corrugator supercilii [21]. In agreement, observing sad faces associated with positive characteristics elicited stronger frowning compared to similar faces associated with neutral characteristics, whereas sad faces associated with negative characteristics even resulted in the opposite pattern of decreased activation [22]. Frowning in response to angry facial expressions shows a different pattern and is also dependent on social context. For example, in competitive situations angry facial expressions may not be mimicked by the observer, since an angry opponent can indicate an advantage for the observer thus reflecting a ‘positive’ signal resulting in relaxation of the corrugator supercilii [10]. However, angry facial mimicry is enhanced in a situation when treated unfair by others, whereas the opposite effect is shown after a fair treatment by others [23]. Such increased angry facial response to unfair others might serve as a corrective signal to enforce norm compliance. Thus, although facial communication by emotional expressions is fast, automatic, and largely beyond voluntary control, the above studies show that it is far from reflexive and can signal variable underlying motives depending on social context.

Considering the importance of adult emotional signaling towards children, it is surprising that dynamical facial EMG responses of adults interacting with children have received only little attention. During early infancy, children’s communication through emotional facial expressions is primarily with their direct caregivers. However, during childhood socialization through facial communication extends to secondary caregivers, teachers, and peers [24]. If facial emotional responses to children’s emotional expressions can help regulate their behavior and contribute to their socialization when growing up, it is of relevance to know the contextual factors that alter these emotional responses towards children. In the current study, we attempted to shed a first light on how contextual information may alter adult emotional expressions towards unknown children. In a within-subject design, participants were given background information on children’s temperament and their domestic situation. Temperament and domestic situation were chosen since these personal factors have predictive value for a child’s social-cognitive development, and socialization by emotional expressions can serve as one of the underlying processes mediating this relation [8, 25]. Pictures of the children were combined with statements of positive or negative behavior and of either a positive or negative domestic situation. Using facial EMG of the corrugator supercilii, the zygomatic major, and the mentalis, we measured the effect of social context information on the imitation of facial expressions of these children displaying happy, sad, and angry facial expressions. We expected that frowning would be elicited strongest towards children characterized by negative behavior when displaying angry facial expressions, possibly functioning as a corrective signal. Such an effect could additionally be mediated by a child’s domestic situation. For smiling towards happy facial expressions, an opposite effect is expected, with enhanced zygomatic major activation towards children characterized by positive behavior. For sad faces, we expected that frowning would be increased towards children with difficult domestic situations displaying positive behavior, as in this context frowning could serve as a marker for empathic responding.

## Methods

### Participants

Sample size was determined based on our previous work in which a small final sample of N = 26 yielded a significant effect of context manipulation in a similar setup [23]. Since small samples can lead to exaggerated estimates of effect size, in the present study we increased our sample to n = 40. Female participants were included in the study and performed a series of three experimental blocks. Only female participants were included since it has been shown that females are more facially reactive than males [26]. EMG data from one participant was not recorded due to technical problems, yielding a final N of 39 (mean age: 21.4 years, S.D.: 2.0, range 18–25). Participants were told that they would perform an emotional learning task during which electrodes would monitor ‘physiological changes’ as a cover story. None of the participants had a history of psychiatric or neurological conditions. Written informed consent was obtained and volunteers received financial compensation or course credits for participation. The study was conducted in accordance with the latest declaration of Helsinki and was approved by the local ethical committee of the faculty of social science, Utrecht University.

### Experimental task and stimuli

The experimental task consisted of three separate blocks. In the first block baseline facial responses towards angry, happy, and sad expressions displayed by four different children were obtained. In the second block, participants learned contextual information about these children. The final block was identical to the first block, which allowed us to investigate the effect of the contextual information on facial mimicry.

#### Block one: baseline facial responses

**Fig 1:**
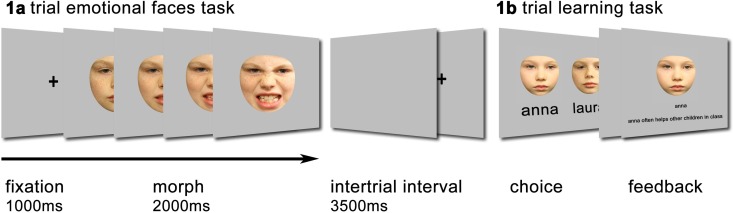
Display of the task trials. Panel A displays a trail of the emotional faces task (block 1) in which a neutral face morphs into an emotional face. Panel B displays a trial of the learning task, in which a participant has to choose among two children to learn context information about these children (block 2). The parent of this model provided written informed consent, as outlined in the PLOS consent form, for publication of their child’s photographs (doi:10.1371/journal.pone.0079131).

Block one consisted of a passive viewing task in which video clips of children’s faces were presented that gradually morphed from a neutral into an emotional expression (Fig 1A). Duration of the clips was 2000 ms, in which the face morphed during the first 1000 ms and remained on the screen in full emotion for an additional 1000 ms. Happy, angry, and sad facial expressions were presented from four different children (2 male, 2 female; 8–9 years old) yielding a total of 12 different stimuli. Pictures of neutral and emotional expressions were taken from the Dartmouth Database of Children's faces [27], and Morphs were created using WinMorph software (www.debugmode.com/winmorph/). The stimuli were each presented three times resulting in a total of 36 trials and were preceded by a 1000 ms fixation cross. Inter-trial interval was fixed at 3500 ms. Before the start of the passive viewing tasks, a neutral facial expression of all four children was shown once, together with a sentence introducing the children with neutral background information (e.g. “This is Anna; she is in third grade and likes to play outside”).

#### Block two: context learning task

For block two we created a learning task in which participants were trained to associate contextual information with the four different children. Participants were given information about the domestic situation of the children, and subsequently learned about the children’s behavior. This procedure resulted in the following four different stimuli conditions: 1) good domestic situation and positive behavior, 2) difficult domestic situation and positive behavior, 3) good domestic situation and negative behavior, 4) difficult domestic situation and negative behavior.

First, neutral pictures of all four children were presented in subsequent order accompanied by a short story (three sentences) describing either a positive or negative domestic situation (example positive: “Anna has a little brother. Together with their parents they do many nice activities. Anna likes it at home and things have gotten even better since they got a dog”; example negative: “Tim is an only child. He doesn’t know his father. Tim’s mother thinks her career is very important and hardly has time for Tim.”). Next, participants learned about the children’s behavior. In this phase, 24 learning trials were shown. At the start of each trial, neutral pictures of two of the four children were presented on the screen, among which the participant had to choose (Fig 1B). After choosing one the two children, the picture of that child was displayed together with a sentence describing either positive or negative behavior (example positive: “Tim takes good care of his little sister”; example negative: “Anna always starts screaming when she is angry”). Faces were quasi-randomly presented to guarantee that all possible combinations of children were shown an equal number of times. Participants were instructed to pay attention to the information as they would be tested afterwards.

To control for interactions between physical characteristics of the children and the stimuli conditions, we created two task versions with different child-condition combinations that were balanced over participants. After the learning phase we checked whether participants had learned the child-context associations with a multiple-choice test. A random sentence from the learning task was presented together with the pictures of all four children, and participants had to match the child to the sentence. Eight of such multiple choice questions were presented, with two questions referring to each child.

#### Block three: effect of context on facial responses

Block three was identical to the first block, only now participants had learned about the domestic environment and behavior of the different children.

### Data reduction and statistical analyses

EMG responses were recorded from bipolar electrode montages from the left zygomaticus major to assess motor responses to happy facial expression, and from the left corrugator supercilii and mentalis muscles (S1 Fig) to measure responses to angry and sad facial expressions [28]. The ground consisted of the active common mode sense and passive driven right leg electrodes (see www.biosemi.com) that were placed midline on the forehead. EMG was recorded at a sampling rate of 2048 Hz using a Biosemi ActiveTwo amplifier and stored for off-line analysis.

Raw EMG traces were 30–500 Hz band pass filtered. For each trial, -1000 ms to +2000 ms response windows were selected time-locked to morph onsets. Baseline correction was applied by subtracting the averaged EMG activity 1000 ms pre-stimulus onset period from the post-stimulus onset values. EMG signals were then rectified and averaged for 250 ms intervals. The resulting 8 time bins were entered into statistical analyses. Data reduction was performed using Brain Vision Analyser 2 (http://www.brainproducts.com/).

For block one, separate 3x8 ANOVAs with emotion (happy, angry, sad) and time (8 bins) as within subject factors were performed for the three muscles to detect mimicry. For the third block, muscle activity of the zygomaticus and the corrugator was entered in two separate 3x2x2x8 ANOVAs with emotion (happy, angry, sad), domestic situation (good, difficult), behavior (positive, negative), and time (8 bins) as within-subject factors. Emotion was included in this analysis to detect mimicry in the third block. In case of the significant interactions or main effects of the factors ‘domestic situation’ and ‘behavior’, the ANOVA was split up in 2x2x8 ANOVAs for the different emotions to further investigate the effect of domestic situation and behavior in the separate emotion conditions. To test the direction of possible effects in these ANOVAs, further post-hoc pairwise comparisons were performed. For all reported F-tests sphericity was tested using Mauchly’s test, and when significant Greenhaus-Geisser correction was applied. All statistical analyses were performed in SPSS 23.

## Results

### Block one: baseline facial responses

**Fig 2:**
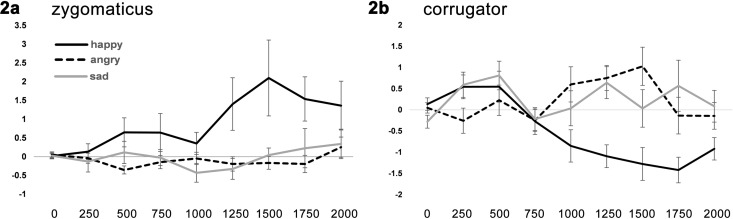
Facial responses during block 1. Zygomaticus major (panel A) and corrugator supercilii (panel B) activation when looking at angy, happy, and sad children’s faces in the first emotional faces task (block 1), before context information was learned. Time is displayed on the x-axis in milliseconds. Muscle activation is displayed on the y-axis in microvolts. Error bars indicate standard error of the mean.

The zygomaticus showed a significant effect of emotion (F(1.10, 41.89) = 5.71, *p* = .019, ɳ^2^ = .13). Post-hoc pairwise comparisons showed that zygomaticus activation towards happy faces differed significantly from activation towards angry (*p* = .027) and sad faces (*p* = .011; see Fig 2A), whereas zygomaticus activation did not differ between sad and angry faces (*p* = .58).

The corrugator showed a significant interaction of emotion x time (F(7.24, 275.15) = 3.44, *p* = .001, ɳ^2^ = .083) and a main effect of emotion (F(1.59, 60.34) = 7.13, *p* = .003, ɳ^2^ = .16). Post-hoc pairwise comparisons showed that the corrugator was more active towards angry and sad faces compared to happy faces (*p* = .005 and *p* = .006), whereas there was no difference between corrugator responses toward sad and angry faces (*p* = .64; see Fig 2A)

For the mentalis, no significant interaction or main effects were observed for the included factors (all *p*’s > .05) suggesting insensitivity to the present experimental manipulation. Therefore, mentalis was discarded from further analyses.

#### Block two: manipulation check

At the end of block two participants performed an 8 item multiple-choice test to assess whether the manipulation (learning phase) had been successful. On average, participants made 1.19 (SD = 1.08) errors with only one participant making a maximum of 4 errors. Chance level equals 6 errors, indicating that the learning phase was successful. To control whether the manipulation was successful in all four conditions similarly, a repeated measures ANOVA on performance in the different categories was run. This analyses revealed no significant differences between any of the categories (all *p*’s > .05), demonstrating that performance was equal in all conditions.

#### Block three: context learning effect on facial responses

To investigate the effect of learned context information on facial responses towards children, we tested the effect of the 4 different conditions on activation of the zygomaticus and corrugator to the three emotional expressions. For the zygomaticus no effect of domestic context or behavior was observed, nor an interaction among these variables or with the factor emotion. There was a significant main effect of time (F(2.41, 91.47) = 3.11, *p* = .041, ɳ^2^ = .08), and of emotion (F(1.22, 46.50) = 4.42, *p* = .034, ɳ^2^ = .10). Post-hoc pairwise comparisons confirmed facial mimicry by showing stronger zygomaticus activation towards smiling compared to angry faces (*p* = .014). This was however similar towards children in all conditions.

For the corrugator, we observed a significant interaction of emotion x domestic situation x behavior (F(1.93, 73.38) = 5.16, *p* = .009, ɳ^2^ = .12), as well as for emotion x time (F(7.65, 290.57) = 2.52, *p* = .013, ɳ^2^ = .06), indicating significant mimicry (stronger corrugator activation towards angry faces compared to happy faces, *p* = .01) in block 3 that differed depending on both contextual factors. To further specify these results, we split out the analysis for the three different emotion conditions.

**Fig 3:**
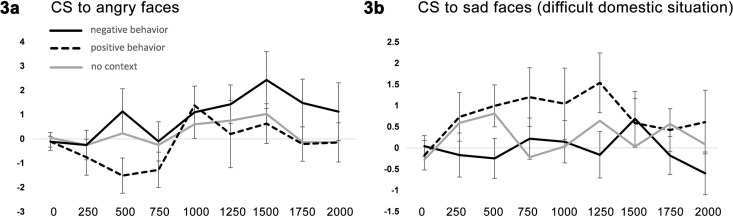
Facial responses during block 3. Activation of the corrugator supercilii (CS) is plotted towards angry faces (panel A) and towards sad faces of children with a difficult domestic situation (panel B) in block 3. The different lines depict activation towards children showing either positive or negative behavior, and towards baseline (block 1). Time is displayed on the x-axis in milliseconds. Muscle activation is displayed on the y-axis in microvolts. Error bars indicate standard error of the mean.

For corrugator activation towards angry facial expressions there was a significant main effect for the factor child behavior (F(1, 38) = 6.34, *p* = .016, ɳ^2^ = .14). Participants displayed stronger corrugator activation towards children associated with negative versus positive behavior (pairwise comparison *p* = .016). There were no main effects of, or interactions with, domestic situation (all *p*’s > .05). To test the positive and negative behavior against the baseline in block one, we collapsed the data over both domestic situations and ran additional ANOVA with baseline corrugator and collapsed corrugator responses towards positively and negatively behaving children. Pairwise comparisons showed that corrugator activity towards negatively behaving children is higher compared to positively behaving children (*p* = .016), and compared to baseline (*p* = .041). Baseline and positively behaving children did not differ significantly (*p* = .28; see Fig 3A).

Next, we investigated the effect of learned information on corrugator responses to sad children’s faces. Here, we found a significant interaction between a child’s behavior, and domestic situation (F(1, 38) = 8.08, *p* = .007, ɳ^2^ = .18). Post-hoc pairwise comparisons revealed a different pattern of muscle activation towards positive and negative behaving children with either a difficult or a good domestic situation. When viewing sad faces of children with a difficult domestic situation, participants showed increased corrugator responses towards children displaying positive compared to negative behavior (*p* = .018; see Fig 3B). When looking at sad faces of children with a positive domestic situation, there was no significant difference between children displaying positive or negative behavior (*p* = .44; Fig 3B). Comparisons of the corrugator responses to sad faces in the third block with the baseline obtained from block one did not reveal significant differences (all *p* > .05)

Finally, we tested the effect of learned information on corrugator responses towards happy facial expressions. This analysis did however not show a significant effect of domestic situation, behavior, or an interaction between these factors (all *p* > .05).

## Discussion

The aim of the current study was to investigate whether contextual information of a child’s temperament and domestic situation would alter automatic facial responses to emotional expressions of children. In the first block of the task we measured baseline mimicking of a child’s emotional expressions. We observed mimicry as indicated by increased activation of the zygomaticus major to happy expressions and increased corrugator supercilii activation to angry and sad facial expressions (Fig 2). In the next block participants successfully learned contextual information about the children; whether children displayed positive or negative behavior, and whether they were exposed to a positive or negative domestic situation. In the final block the effect of this learned information on facial responses to the children’s emotional expressions was measured. Contextual information did not affect mimicry of happy facial expressions of children, but did alter the responses to angry and sad expressions. Participants showed increased corrugator supercilii activation towards angry facial expressions of children behaving negatively compared to those behaving positively, as well as compared to baseline. There was no effect of domestic situation on facial responses to angry children’s faces (Fig 3A). Towards sad faces, corrugator supercilii activation in response to positively and negatively behaving children differed depending on the learned domestic situation. In children with a negative domestic situation, there was increased corrugator supercilii activation towards children showing positive versus negative behavior, while no significant difference between positively and negatively behaving children was seen in a positive domestic situation (Fig 3B).

Overall these data demonstrate that automatic facial responses towards children’s emotional expressions are indeed significantly affected by the observer’s knowledge of child’s personal factors. Increased corrugator supercilii activation towards angry children of which observers have learned that they behave negatively could serve as a corrective or punitive signal, similar to increased corrugator supercilii activation observed towards adults after being treated unfair [23]. In contrast, increased activation of the corrugator supercilii towards sad faces is more likely to signal empathy, which is reflected in increased activation of this muscle towards sad children displaying positive compared to negative behavior, an interpretation in line with earlier observations [22]. Importantly, in the current study this effect was observed only towards children with a negative domestic situation, not towards children with a positive domestic situation. In line with the above interpretation of frowning responses to sad faces [19, 21], this might be caused by increased sympathy for children who both look sad and have a difficult situation at home. Nonetheless, the underlying motives of participants were not addressed in this study, and our functional interpretation of altered corrugator responses towards angry and sad faces should therefore be empirically tested in future research. We base our interpretations on research showing that corrugator responses towards sadness inducing stimuli are predictive of empathic behavior [20], whereas similar corrugator responses towards angry faces have been observed after unfair versus fair opponents in a trust game [23]. However, irrespective of the underlying motive of the sender, research on the socialization of children clearly demonstrates that emotional expressions of caregivers are important in guiding their behavior [8]. Thus even when arising from a different motivation than norm-enforcement, it can certainly serve such a function from the perspective of the receiving child.

In contrast to the corrugator supercilii, activation of the zygomaticus major in response to happy children’s faces did not differentiate depending on context information. Although this is in contrast to facial responses towards adult faces in which stronger zygomatic major activation was measured towards positive compared to neutral or negative characters [22], an overview of the literature shows that mimicry of happy faces is rather robust to contextual manipulations [10]. Reciprocating a smile is a low-cost response that can signal affiliative intent and smoothen social interactions, and is therefore unlikely to be suppressed. Moreover, previous research has focused mostly on adult faces, and while adult smiles can also indicate dominance motives [29] this is unlikely to be the case for children’s smiles.

The current findings can lead to a better understanding of the dynamic communicative processes that take place between adults and children with diverse backgrounds and behavior, such as in an educational context. Childhood temperament has shown to be predictive for the relation that a child has with its teacher, with a difficult temperament resulting in more conflict [30]. Depending on the teachers’ social skills the child-teacher relationship can be predictive of social, emotional, and educational outcomes [31]. Domestic situation is also a well-known predictive factor for social-emotional and cognitive developmental outcomes [25]. For example, DeGarmo et al. [32] showed that social-economic status predicted parenting behavior, and subsequently, educational achievement. These relations between domestic social-economic status and educational outcome can partly be caused by the expectations teachers have of these children [33]. Thus, both child temperament and a child’s domestic situation can contribute to the quality of the relation they have with their teacher, altering the trajectory of their social-cognitive development. Our findings show that these processes might already take place at the level of automatic facial communication. Furthermore, childhood temperament also contributes in the variation in parenting styles [34], and our current findings might thus hold additional relevance in the context of child-parent interaction. Since parental use of emotional expressions is important in guiding children’s behavior and in the development of socialization [8], it might be of interest to investigate how automatic dynamic facial communication between parents and children is affected by contextual factors. To our knowledge, such studies have not yet been performed. Furthermore, since the current sample consisted of only women, we cannot be certain that similar effects are to be found in males presented with children’s faces. Gender differences in facial mimicry have been reported previously in response to adult faces [35], therefore the question whether males display similar dynamic modulation of mimicry as currently presented should be addressed in future studies.

In sum, in this first study which investigates the modulatory role of contextual information on automatic facial responses towards children, we show modulation of corrugator supercilii activation towards children’s angry and sad faces depending on childhood temperament and domestic situation. This modulation of facial responses might signal corrective responses towards angry children with difficult temperament and empathic responding towards sad children with a difficult domestic situation.

## Supporting Information

S1 Fig Facial muscles. Display of the facial muscles included in the experiment and measured using EMG.
(TIF)

S1 File EMG data file. File including all data described in the paper.
(SAV)
