# Supplementary figures and images for: Social Context Modulates Facial Imitation of Children’s Emotional Expressions
Source: PLoS One. 2016 Dec 8;11(12):e0167991. doi: 10.1371/journal.pone.0167991 (PMC5145233; doi:10.1371/journal.pone.0167991)

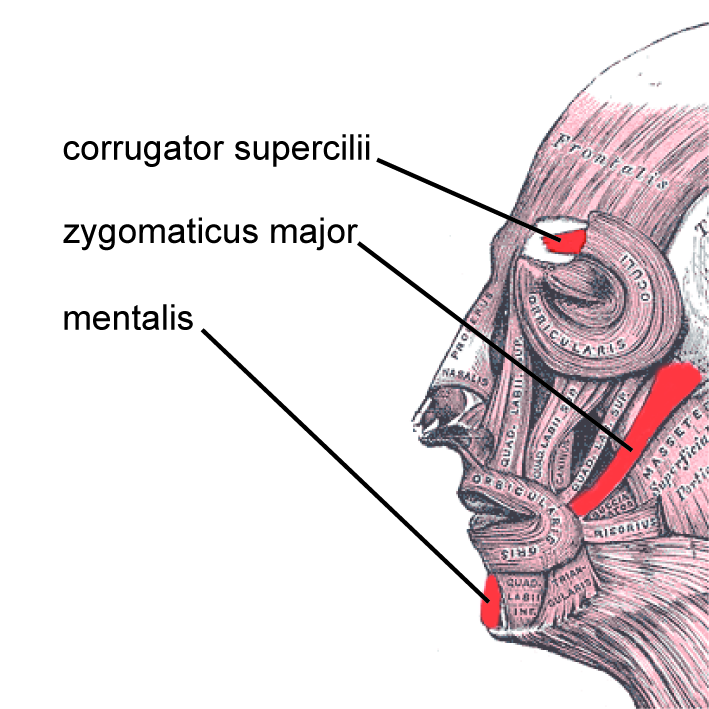

Supplement: S1 Fig — Display of the facial muscles included in the experiment and measured using EMG. (TIF) [file pone.0167991.s001.tif]
